# Supplementary material for: Chemical, Cytotoxic, and Anti-Inflammatory Assessment of Honey Bee Venom from Apis mellifera intermissa
Source: Antibiotics (Basel). 2021 Dec 10;10(12):1514. doi: 10.3390/antibiotics10121514 (PMC8698958; doi:10.3390/antibiotics10121514)
Supplement: Supplementary file 1 [file antibiotics-10-01514-s001.zip › antibiotics-1474263-supplementary.pdf]

Table S1. One-way analysis of variances (ANOVA) for HBV chemical characterization and biological activities outputs (*n* = 5).

Variables	Factor-Regions	Mean	SD	*F*(2.12)	*p*
***In vitro* cytotoxic activity (GI_{50} in µg/mL)**					
**HepG2**	NE	3.87	1.12	3.609	0.059
	C	3.56	0.88		
	S	7.36	4.05		
**NCI-H460**	NE	6.93	2.37	2.960	0.090
	C	7.15	1.74		
	S	10.96	4.16		
**HeLa**	NE	3.67	0.99	3.792	0.053
	C	4.29	0.91		
	S	6.15	2.17		
**MCF-7**	NE	4.03	0.81	5.252	0.023*
	C	5.73	1.12		
	S	8.11	3.16		
**MM127**	NE	3.44	0.79	5.126	0.025*
	C	4.45	0.66		
	S	5.89	1.83		
**PLP2**	NE	13.85	8.64	0.982	0.40
	C	13.79	1.58		
	S	19.95	10.68		
**Anti-inflammatory activity (IC_{50} in µg/mL)**					
**RAW264.7**	NE	4.98	0.72	6.673	0.01*
	C	6.83	0.92		
	S	10.74	4.24		
**Chemical characterization by LC-DAD-ESI/MS^n (µg/mL)**					
**Apamin**	NE	2.05	0.14	1.137	0.35
	C	1.99	0.09		
	S	1.80	0.43		
**PLA2**	NE	7.66	1.13	4.025	0.046*
	C	6.17	1.54		
	S	5.14	1.50		
**Melittin**	NE	71.71	5.06	0.561	0.585
	C	72.53	1.70		
	S	71.68	8.60		
**Metal content**					
**Ca (mg/g)**	NE	1.90	0.83	8.42	0.00*
	C	2.96	0.97		
	S	1.01	0.22		
**Zn (mg/g)**	NE	1.14	0.15	3.63	0.05*
	C	1.27	0.20		
	S	1.01	0.06		
**K (mg/g)**	NE	2.87	0.51	6.93	0.01*
	C	2.38	0.67		
	S	1.69	0.16		
**Na (mg/g)**	NE	1.34	0.22	6.52	0.01*
	C	1.43	0.22		
	S	0.82	0.05		
**Mg (mg/g)**	NE	0.43	0.11	4.82	0.02*
	C	0.43	0.12		
	S	0.27	0.02		
**Pb (µg/g)**	NE	7.19	1.82	5.64	0.01*
	C	4.27	0.69		
	S	4.59	1.73		

*Significant differences

Table S2. Geographic coordinates of the apiaries used for sample collection

Samples	Coordinates
**NE1**	34°00'01.5"N 2°00'48.5"W
**NE2**	34°19'55.7"N 2°07'48.0"W
**NE3**	34°02'40.4"N 1°50'55.7"W
**NE4**	34°47'52.7"N 2°30'08.3"W
**NE5**	34°59'48.0"N 2°23'35.7"W
**C1**	33°52'15.1"N 6°21'04.4"W
**C2**	33°53'48.3"N 6°17'54.8"W
**C3**	33°54'18.6"N 6°01'38.6"W
**C4**	32°32'05.7"N 6°22'31.5"W
**C5**	32°08'38.1"N 6°31'30.5"W
**S1**	30°37'37.3"N 5°27'17.6"W
**S2**	29°45'57.9"N 7°59'22.0"W
**S3**	29°39'58.2"N 7°58'55.6"W
**S4**	30°25'13.2"N 5°53'03.2"W
**S5**	30°17'30.2"N 5°48'41.5"W

NE – northeast; C – center; S – Southern Morocco
